# Systematic Evaluation of Aegle marmelos-Derived Compounds: Potential Therapeutic Agents Against Inflammation and Oxidative Stress

**DOI:** 10.7759/cureus.57499

**Published:** 2024-04-03

**Authors:** Hota Sankirtha, Logalakshmi Thirumani, Arockia Alex, Brahma Neha, Sugumar Vimal, Inamul Hasan Madar

**Affiliations:** 1 Biochemistry, Saveetha Medical College and Hospital, Saveetha Institute of Medical and Technical Sciences (SIMATS), Chennai, IND; 2 Multiomics and Precision Medicine Laboratory, Center for Global Health Research, Saveetha Medical College and Hospital, Saveetha Institute of Medical and Technical Sciences (SIMATS), Chennai, IND

**Keywords:** anti-oxidant, anti-inflammatory, pharmacokinetic properties, molecular docking, aegle marmelos

## Abstract

Aim

This study aimed to evaluate the potential antioxidant and anti-inflammatory properties of *Aegle marmelos* active compounds through a multifaceted approach. The investigation encompasses molecular docking studies, computational pharmacokinetic predictions, and in vitro assessments, with a focus on understanding their physiochemical properties, pharmacokinetics, and molecular interactions.

Materials and methods

This study was conducted in the Research Department of Biochemistry, Saveetha Medical College & Hospital, Saveetha Institute of Medical and Technical Sciences (SIMATS), Tamilnadu, India. The study employed Soxhlet and methanol extraction techniques to obtain *Aegle marmelos* extracts, which were then subjected to antioxidant and anti-inflammatory assays. Antioxidant activity was assessed using the H_2_O_2_ assay, while anti-inflammatory potential was determined via the egg albumin denaturation assay. Molecular docking studies were conducted with human heme oxygenase 1 (HO-1) and human zanthine oxidoreductase (XO) proteins to elucidate potential therapeutic interactions. Furthermore, computational tools like SwissADME, pkCSM, and ADMETlab 2.0 were utilized to predict physiochemical and pharmacokinetic properties, providing insights into the compound absorption, distribution, metabolism, and excretion profiles. This integrated approach aimed to comprehensively evaluate the therapeutic potential of *Aegle marmelos*-derived compounds against inflammation and oxidative stress-related disorders, paving the way for future drug development endeavors.

Results

In the antioxidant assay, *Aegle marmelos* methanolic tuber extracts showed exceptional absorption of 87.4%, surpassing the reference standard. In the anti-inflammatory assay, the extracts displayed an absorption of approximately 79%, indicating significant anti-inflammatory potential. Auraptene, imperatorin, luvangetin, and psoralen exhibited favorable pharmacokinetic properties and adherence to the Lipinski rule of 5, suggesting promising drug development potential. In molecular docking, imperatorin demonstrated the highest binding affinity to HHO-1 and XO.

Conclusion

The study on *Aegle marmelos* highlights its potential as a therapeutic agent due to its potent antioxidant and anti-inflammatory properties. Phytochemical constituents, such as auraptene, imperatorin, luvangetin, and psoralen, show promising pharmacokinetic profiles, suggesting their suitability for drug development. Molecular docking analysis reveals imperatorin as the most effective binder to key enzymes, emphasizing its therapeutic potential against inflammation and oxidative stress-related disorders.

## Introduction

Anti-inflammatory and antioxidant mechanisms are crucial for maintaining health and preventing a wide range of diseases. Inflammation is indeed a fundamental physiological response by the body's immune system to remove harmful stimuli, including damaged cells, irritants, or pathogens, and initiate the healing process. Inflammation is the body's defense against harm but can lead to diseases, like arthritis, cardiovascular disease, and diabetes, if it becomes chronic. Persistent inflammation damages tissues, contributing to these conditions by disrupting normal cellular functions and responses [1]. Similarly, oxidative stress, resulting from an imbalance between the production of reactive oxygen species (ROS) and the body's ability to neutralize them, contributes to cellular damage and has been implicated in aging, neurodegeneration, and cancer [2]. Anti-inflammatory agents function by inhibiting the synthesis or action of pro-inflammatory mediators, such as cytokines and prostaglandins, modulating the activity of various immune cells and blocking critical signaling pathways like NF-κB involved in inflammatory responses. Concurrently, antioxidants counteract oxidative stress by directly scavenging ROS, chelating catalytic metal ions, and bolstering the body's endogenous antioxidant defense systems, including enzymes, like superoxide dismutase and glutathione peroxidase.

Human heme oxygenase 1 (HO-1), an inducible enzyme, plays a critical role in the degradation of heme into biliverdin, free iron, and carbon monoxide (CO) [3]. This process has significant anti-inflammatory implications. The products of heme degradation by HO-1 have been shown to exert various protective effects, including anti-inflammatory, antioxidant, and anti-apoptotic activities. Biliverdin and its reduced form, bilirubin, are potent antioxidants, while CO has anti-inflammatory effects through the modulation of inflammatory cytokine production and the inhibition of the expression of pro-inflammatory genes. XOR is a molybdenum-containing enzyme that plays a dual role in the metabolism of purines, functioning either as xanthine dehydrogenase (XDH) or xanthine oxidase (XO), depending on its form [4]. While XOR's primary function is in purine degradation, leading to the production of uric acid, the XO form contributes to oxidative stress by generating superoxide radicals. Interestingly, XOR inhibitors have been explored for their antioxidant potential, aiming to reduce the production of ROS and mitigate oxidative stress.

In recent years, there has been a growing interest in exploring the therapeutic potential of natural products due to their abundance of bioactive compounds with diverse pharmacological activities [5]. The dual functionality of natural compounds, particularly phytochemicals, such as flavonoids and terpenoids, in exhibiting both anti-inflammatory and antioxidant properties, positions them as promising candidates for therapeutic interventions. *Aegle marmelos*, commonly known as Bael, is a species of deciduous tree indigenous to the Indian subcontinent and Southeast Asia [6]. It holds a venerable position in Ayurveda, Siddha, and other traditional medicine systems across the Indian subcontinent, where it has been used for thousands of years. The holistic utility of the plant, encompassing its leaves, bark, roots, fruits, and seeds, underscores its integral role in phytotherapy. The therapeutic potential of *Aegle marmelos*, particularly its flowers, is an area of increasing scientific interest due to the diverse pharmacological activities they exhibit. *Aegle marmelos* flowers contain a rich profile of bioactive compounds that contribute to their medicinal properties. Research has identified various phytochemicals in flowers, including flavonoids, terpenoids, and essential oils, which play a significant role in their therapeutic applications [7]. These activities include, but are not limited to, antidiabetic, anticancer, antipyretic, analgesic, antimicrobial, and antiviral effects, showcasing the comprehensive pharmacological profile. 

The objective of this study is to systematically evaluate the antioxidant and anti-inflammatory activities of the methanolic extract of *Aegle marmelos*, leveraging both in vitro assays and molecular docking studies. This research aims to fill the critical gap in scientific knowledge regarding the extract’s mechanistic effects on molecular targets involved in inflammation and oxidative stress. By conducting standardized in vitro assays, the study will quantify the extract's capacity to neutralize free radicals and modulate inflammatory markers, thus elucidating its potential therapeutic applications in conditions characterized by oxidative stress and chronic inflammation. Molecular docking studies will be employed to predict the interactions between specific bioactive compounds within the extract and key protein targets in the antioxidant and anti-inflammatory pathways. In addition, the study will incorporate ADME (absorption, distribution, metabolism, and excretion) prediction analyses to assess the pharmacokinetic properties of the identified bioactive compounds, ensuring their potential for effective and safe therapeutic use. This dual approach aims to provide a comprehensive understanding of the molecular basis underlying the extract’s bioactivity, supporting the development of novel natural products for health conditions associated with inflammation and oxidative damage. Ultimately, this research seeks to scientifically validate the traditional uses of *Aegle marmelos*, contributing to the discovery of potent, natural anti-inflammatory and antioxidant agents.

## Materials and methods

This study was conducted in the Research Department of Biochemistry, Saveetha Medical College & Hospital, Saveetha Institute of Medical and Technical Sciences (SIMATS), Tamilnadu, India.

Preparation of plant extract

*Aegle marmelos* leaf powders were purchased from the local alternative medicine practitioner shop in Poonamallee, Chennai, India. For the extraction process, 50 g of the powdered sample was mixed with 250 ml of methanol and subjected to agitation on an orbital shaker at a speed of 190-220 rpm for 48 hours. The resulting mixture was separated, and the supernatant was collected. To obtain a purified extract, the supernatant was passed through a Whatman No. 1 filter paper and concentrated using a rotary flask evaporator at a specific temperature determined by the solvent system employed.

Extraction of the samples

The dried samples were individually ground into powder using a grinder. A predetermined amount of the powdered samples was loaded into a thimble and placed inside the Soxhlet apparatus. The hot Soxhlet extraction method was employed for sequential extraction. The apparatus was operated for 72 hours until the solvent displayed a colored appearance in the siphon, indicating complete extraction of the crude extracts from the samples. The solvents were then evaporated in a rotary vacuum evaporator at 65°C under reduced pressure. The resulting extracts were subsequently dried in a water bath. To preserve their integrity, the dried extracts were carefully sealed in sterilized culture tubes with a capacity of 20 mL and stored in a refrigerator at 2-8°C for subsequent analysis.

Antioxidant activity: H_2_O_2_ assay

All solutions were prepared freshly. One mL of the reaction mixture contained 100 mL of 28 mM 2-deoxy-2-ribose (dissolved in phosphate buffer, pH 7.4) and 500 mL of solutions of various concentrations (10, 20, 30, 40, and 50 mL) 200 µL of 200 µM Fecl3 and 1.04 µM ethylenediaminetetraacetic acid (EDTA) (1:1 v/v), 100 µL H_2_O_2_ (1.0 mM), and 100 µL ascorbic acid (1.0 mM). After an incubation period of one hour at 37 °C, the extent of deoxyribose degradation was about 532 nm against the blank solution.

Anti-inflammatory activity: egg albumin denaturation assay

The anti-inflammatory activity of *Aegle marmelos* was evaluated following the protocol proposed by Muzushima and Kabayashi, with some modifications as mentioned in the study by Das (2019) [8]. A volume of 0.05 mL of various concentrations (10, 20, 30, 40, and 50 µL) of the gel was added to 0.45 mL of bovine serum albumin (1% aqueous solution), and the pH of the mixture was adjusted to 6.3 using a small amount of 1N hydrochloric acid. These samples were incubated at room temperature for 20 minutes, followed by heating at 55°C in a water bath for 30 minutes. After cooling, the absorbance of the samples was measured spectrophotometrically at 660 nm. Diclofenac sodium was used as the standard reference. Dimethylsulfoxide (DMSO) was used as a control. The percentage of protein denaturation was calculated using the following equation: % inhibition = (absorbance of control - absorbance of sample) / absorbance of control × 100.

Protein preparation

**Figure 1:**
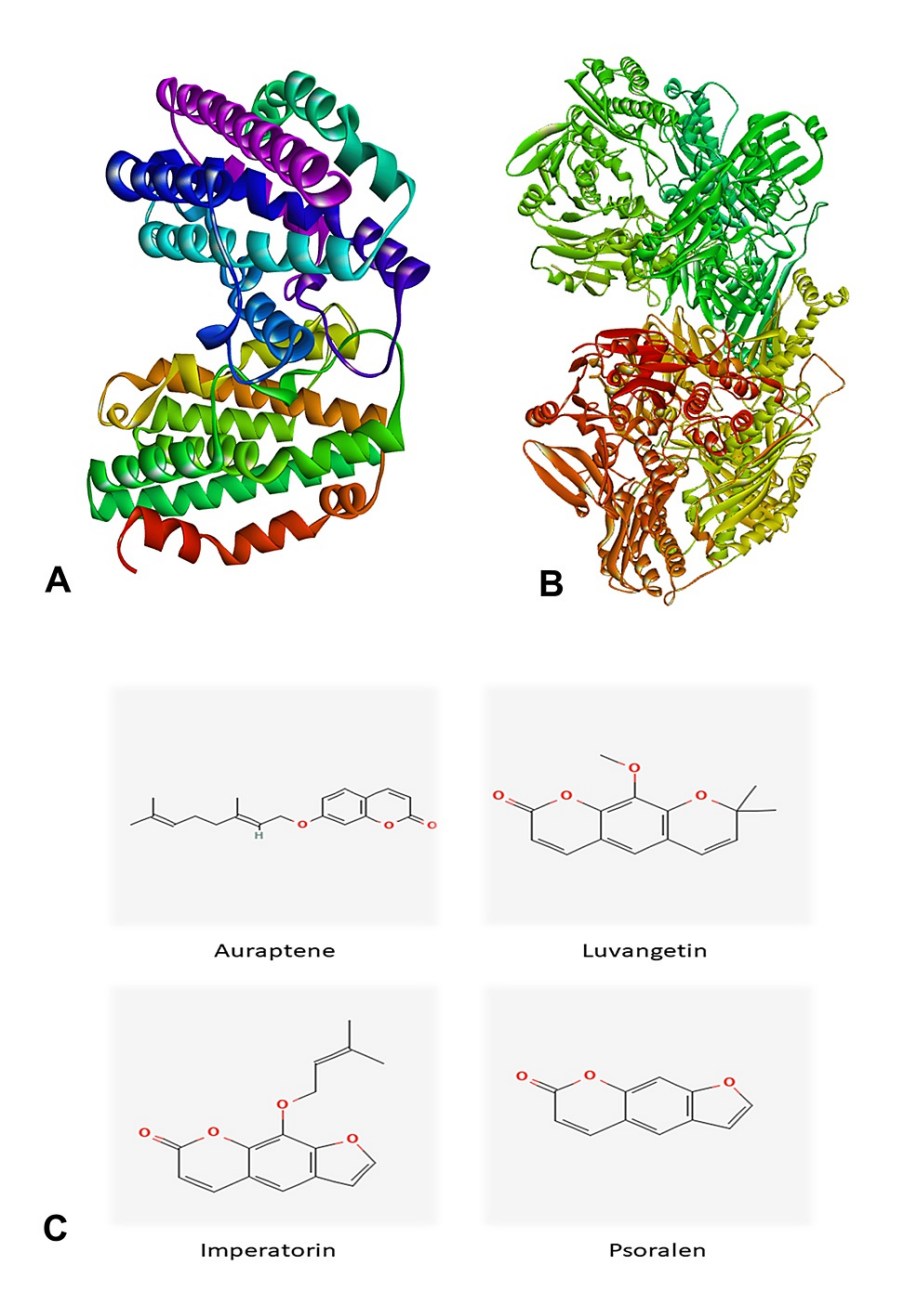
Three-dimensional structure of the protein and ligand (A) 3D-prepared human heme oxygenase 1 (HO-1) (PDB ID: 1N3U). (B) 3D-prepared human xanthine oxidoreductase (PDB ID: 2E1Q). (C) 2D structure of ligand molecules. (Image retrieved from the Protein Data Bank (PDB) and PubChem web tools)

The crystal structures of HO-1 (PDB ID: 1N3U) [9] and human xanthine oxidoreductase (PDB ID: 2E1Q) [10] were obtained from the Research Collaboratory for Structural Bioinformatics (RCSB) database with resolutions of 2.58 Å and 2.60 Å, respectively. Following retrieval, the structures underwent preprocessing steps, including the removal of complexes bound to the protein receptor molecules and the determination of ionizable residue protonation states to ensure accurate electrostatic interactions during docking. To streamline the system, water molecules and extraneous ligands were eliminated from the protein structures. Force field parameters were then applied to mimic the protein's behavior during docking simulations. Subsequently, optimization and refinement procedures were conducted on the protein structures before initiating molecular docking studies (Figures 1A, 1B).

Ligand preparation

Compounds from *Aegle marmelos* leaf, such as aurapten, imperatorin, psoralen, and luvangetin, were used as ligand molecules (Figure 1C). The molecular structures of these compounds were retrieved from PubChem [11] (http://pubchem.ncbi.nlm.nih.gov). Utilizing Chimera [12], the molecular structures, initially in the SDF format, were converted into PDB structures for a more detailed examination of their three-dimensional arrangements.

Pharmacokinetic prediction

The compound's ADME properties were predicted using SwissADME (http://www.swissadme.ch/) [13], pkCSM (http://biosig.lab.uq.edu.au/pkcsm) [14], and ADMETlab 2.0 (https://admetmesh.scbdd.com/) [15]. SwissADME provides a comprehensive evaluation of lead molecules, focusing on pharmacokinetics, drug likeliness, and medicinal chemistry friendliness, while pkCSM emphasizes the compound's pharmacokinetic profile, considering absorption, distribution, metabolism, and excretion properties. ADMETlab further enhances the assessment by providing insights into additional ADMET properties crucial for determining the interplay between pharmacokinetics, toxicity, and potency. This integrated approach ensures a thorough understanding of the compound's suitability for further drug development, highlighting the importance of ADMET properties in mitigating clinical trial failures.

Molecular docking

The process of docking the ligand against the protein involves utilizing Autodock [16], where various binding conformations and interactions are examined. Subsequently, a scoring function is employed to assign a score to the best-docked ligand complex selected from the analyses.

Analysis of docked complexes

Following the docking procedure, the docking data were analyzed to identify the most promising selections for further investigation. This analysis includes assessing the estimated interaction energy to determine each ligand's binding affinity and organizing the ligands based on their binding affinity scores. The Biovia Discovery Studio visualizer (2021) was utilized to identify hydrogen bonds, hydrophobic interactions, and electrostatic interactions between ligands and proteins within the docked structures. These interactions serve to elucidate ligand mechanisms and facilitate structural optimization.

## Results

In vitro antioxidant activity

**Figure 2:**
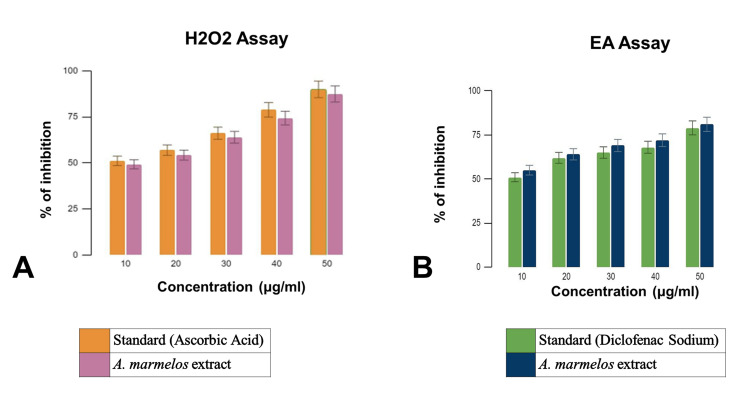
In vitro assessment of methanolic extract from Aegle marmelos (A) Antioxidant characteristics of the methanolic extract derived from Aegle marmelos. (B) Anti-inflammatory characteristics of the methanolic extract derived from Aegle marmelos.

In the antioxidant assay, it was observed that 50 μL of *Aegle marmelos* methanolic tuber extracts exhibited an exceptional absorption percentage of approximately 87.4%. This remarkable result surpassed that of the reference standard, indicating the potent antioxidant activity of the extracts. The high absorption percentage underscores the effectiveness of *Aegle marmelos* in scavenging free radicals and mitigating oxidative stress. These findings highlight the potential therapeutic significance of *Aegle marmelos* as a natural antioxidant agent. Figure 2A illustrates the antioxidant characteristics of the methanolic extract derived from *Aegle marmelos*.

In vitro anti-inflammatory response

In the anti-inflammatory assay, it was determined that 50 μL exhibited an absorption percentage of approximately 79%. This finding underscores the significant anti-inflammatory potential of the tested sample. When compared to the reference standard, the observed absorption percentage highlights the efficacy of the sample in mitigating inflammatory responses. The substantial absorption percentage suggests that the tested sample possesses notable anti-inflammatory properties, indicating its potential therapeutic relevance in combating inflammation-related conditions. Figure 2B illustrates the antioxidant characteristics of the methanolic extract derived from *Aegle marmelos*.

Physiochemical properties

**Figure 3:**
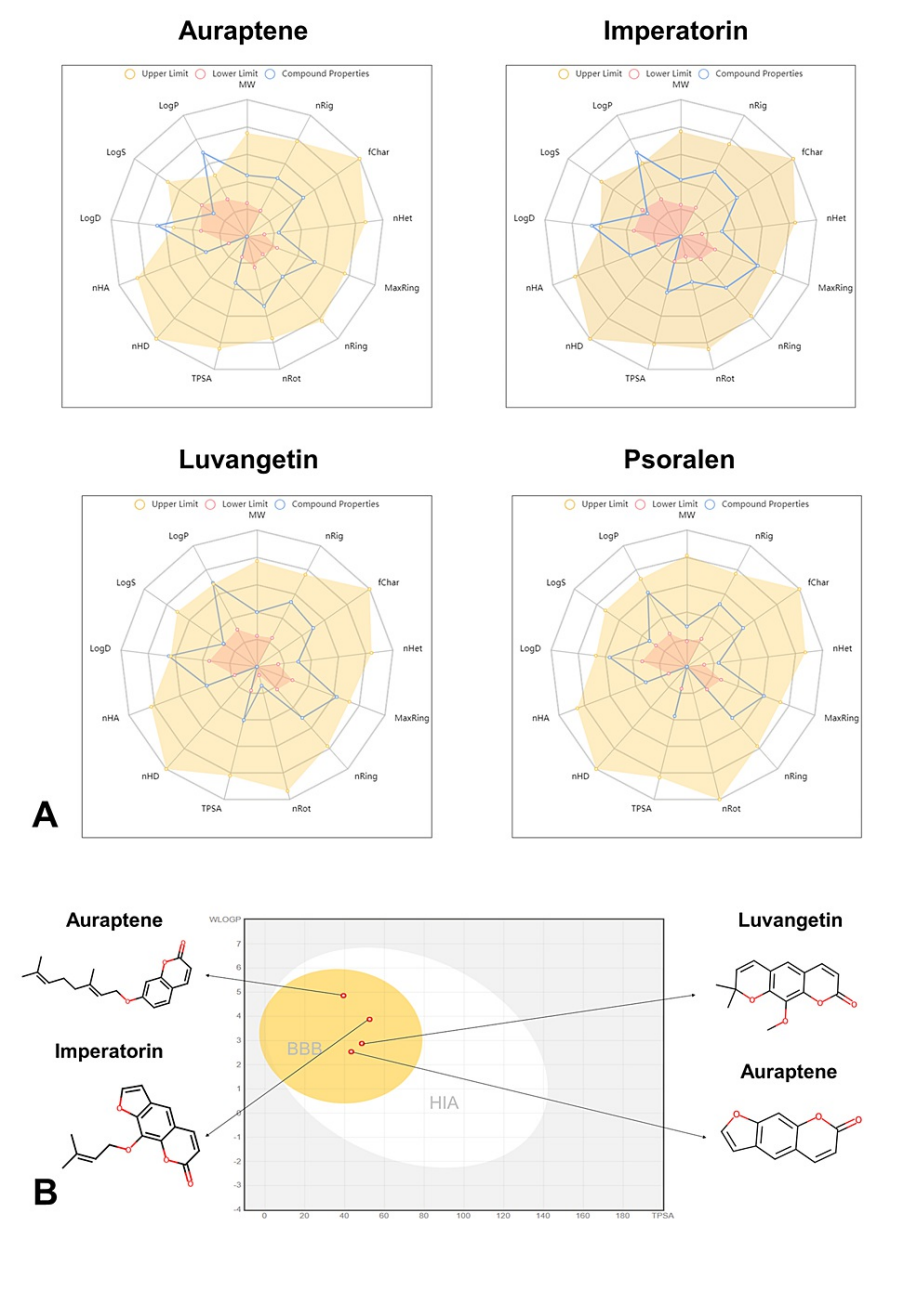
(A) Bioavailability radar of ligand molecules. (B) BOILED-Egg graph of the lead molecules (x-axis: TPSA Å², y-axis: WLOGP) MW: molecular weight, nRig: number of rings, fChar: formal charge, nHet: number of heteroatoms, MaxRing: number of atoms in the biggest ring, nRing: number of rigid bonds, nRot: number of rotatable bonds, TPSA: topological polar surface area, nHD: number of hydrogen bond donor, nHA: number of hydrogen bond acceptor, LogD: LogP at physiological pH 7.4, LogP: log of the octanol/water partition coefficient, LogS: log of the aqueous solubility. Image generated using SwissADME and ADMElab 2.0 software.

Auraptene, with a molecular weight of 298.38 g/mol and 22 heavy atoms, exhibits moderate size and complexity. It possesses 10 aromatic heavy atoms and six rotatable bonds, indicating a degree of flexibility. With three hydrogen bond donors and no hydrogen bond acceptors, auraptene may engage in moderate intermolecular interactions. Its total polar surface area (TPSA) of 39.44 Å² suggests the limited polar surface area available for interactions. Imperatorin, with a lower molecular weight of 270.28 g/mol and 20 heavy atoms, demonstrates similar structural features to auraptene but with a higher number of aromatic heavy atoms (13). It has fewer rotatable bonds (three) and more hydrogen bond donors (four). Luvangetin and psoralen, with molecular weights of 258.27 g/mol and 186.16 g/mol respectively, exhibit fewer heavy atoms, aromatic heavy atoms, and rotatable bonds compared to auraptene and imperatorin. Luvangetin and psoralen both have four hydrogen bond donors. The TPSA values for luvangetin (48.67 Å²) and psoralen (43.35 Å²) suggest a slightly larger polar surface area compared to auraptene and omperatorin (Table 1). Figure 3A depicts the bioavailability radars for four compounds: auraptene, imperatorin, luvangetin, and psoralen. Figure 3B depicts the human intestinal absorption and blood-brain barrier (BBB) penetration of the compound molecules.

**Table 1:** Physiochemical properties of ligand molecules TPSA: topological polar surface area, LogD: LogP at physiological pH 7.4, LogP: log of the octanol/water partition coefficient, LogS: log of the aqueous solubility

Physiochemical Properties	Auraptene	Imperatorin	Luvangetin	Psoralen
Molecular weight	298.38 g/mol	270.28 g/mol	258.27 g/mol	186.16 g/mol
Heavy atoms	22	20	19	14
Aromatic heavy atoms	10	13	10	13
Rotatable bonds	6	3	1	0
H-bond donor	3	4	4	3
H-bond acceptor	0	0	0	0
TPSA	39.44 Å²	52.58 Å²	48.67 Å²	43.35 Å²
logS	-5.577	-4.580	-3.962	-3.433
logP	5.868	3.879	3.070	2.252
logD	4.186	3.532	3.103	2.406

Pharmacokinetic properties

Auraptene, imperatonin, luvangetin, and psoralen demonstrate favorable pharmacokinetic properties conducive to drug development. With high human intestinal absorption (HIA) percentages ranging from 95.416% to 97.755%, these compounds exhibit efficient absorption in the gastrointestinal tract, suggesting favorable bioavailability. In addition, their moderate to high CaCo-2 permeability values (ranging from 1.295 to 1.634) further support their efficient absorption. Despite showing low to moderate permeability across the BBB with logBB values ranging from 0.129 to 0.472 and poor to moderate permeability into the central nervous system (CNS) with logPS values ranging from -2.556 to -1.714, these characteristics may be advantageous in avoiding potential central nervous system-related adverse effects. Overall, the pharmacokinetic properties of these compounds suggest promising drug development potential, with efficient gastrointestinal absorption and limited penetration into the central nervous system (Table 2). 

**Table 2:** Pharmacokinetic properties of ligand molecules HIA: human intestinal absorption, Caco_2_: human epithelial colorectal adenocarcinoma cells, BBB: blood-brain barrier, CNS: central nervous system, VDss: volume of distribution in steady state

Compound name	Absorption		Distribution			Excretion
	HIA (%)	CaCO_­2_ (log Papp in 10^-6^ cm/s)	BBB (logBB)	CNS (logPS)	VDss (log L/Kg)	Clearance (log ml/min/kg)
Auraptene	95.416	1.634	0.129	-1.88	0.491	1.083
Imperatonin	97.755	1.383	0.176	-1.905	0.147	0.994
Luvangetin	97.681	1.31	0.472	-2.556	0.108	0.829
Psoralen	96.668	1.295	0.41	-1.714	-0.13	0.773

Molecular docking

**Figure 4:**
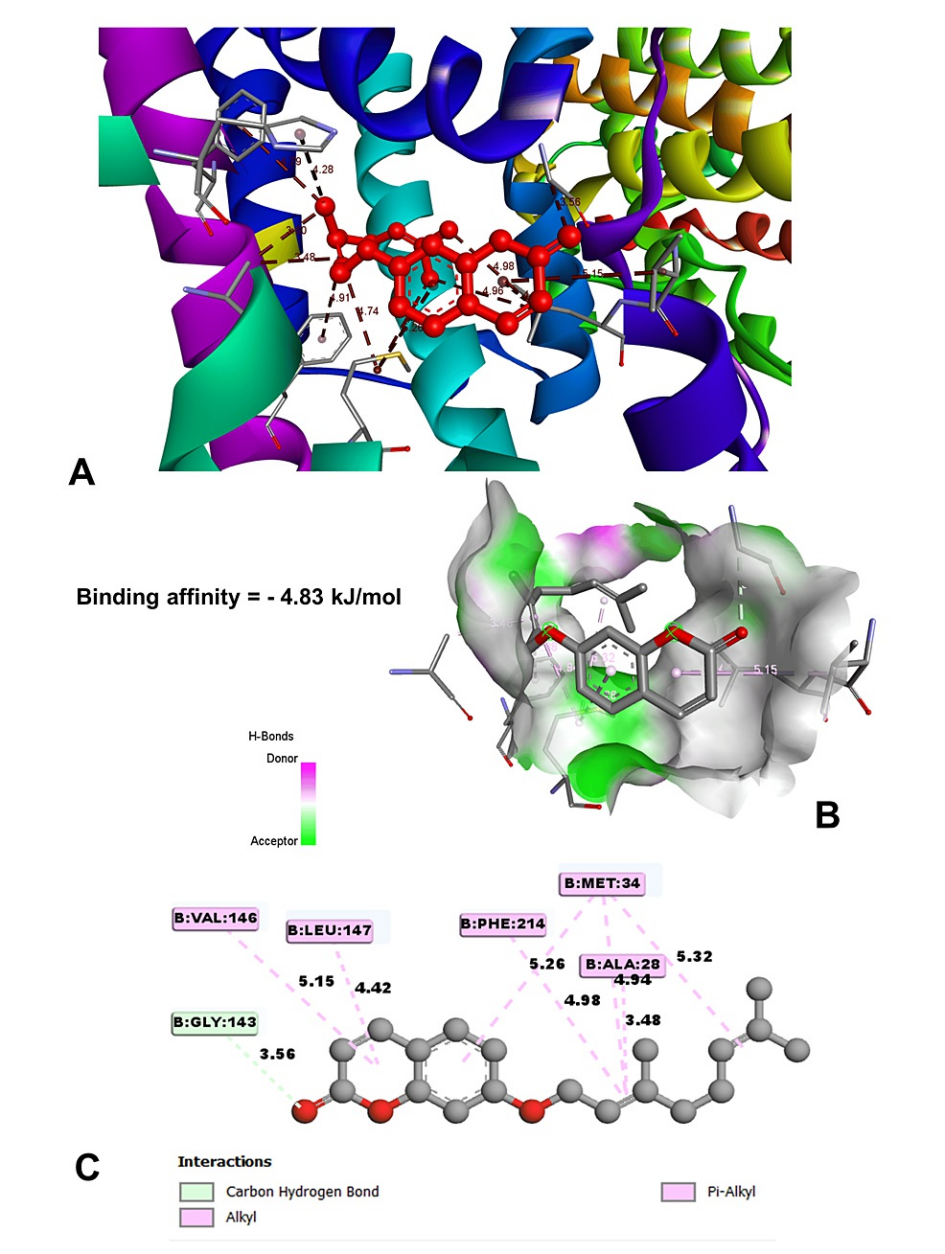
Binding affinities between the auraptene molecule and HO-1 receptor (A) Localization of auraptene binding within heme oxygenase 1 (HO-1). (B) Three-dimensional interaction between auraptene and its receptor. (C) Two-dimensional interaction between auraptene and its receptor. (Image generated by BIOVIA Discovery Studio and Chimera software)

**Figure 5:**
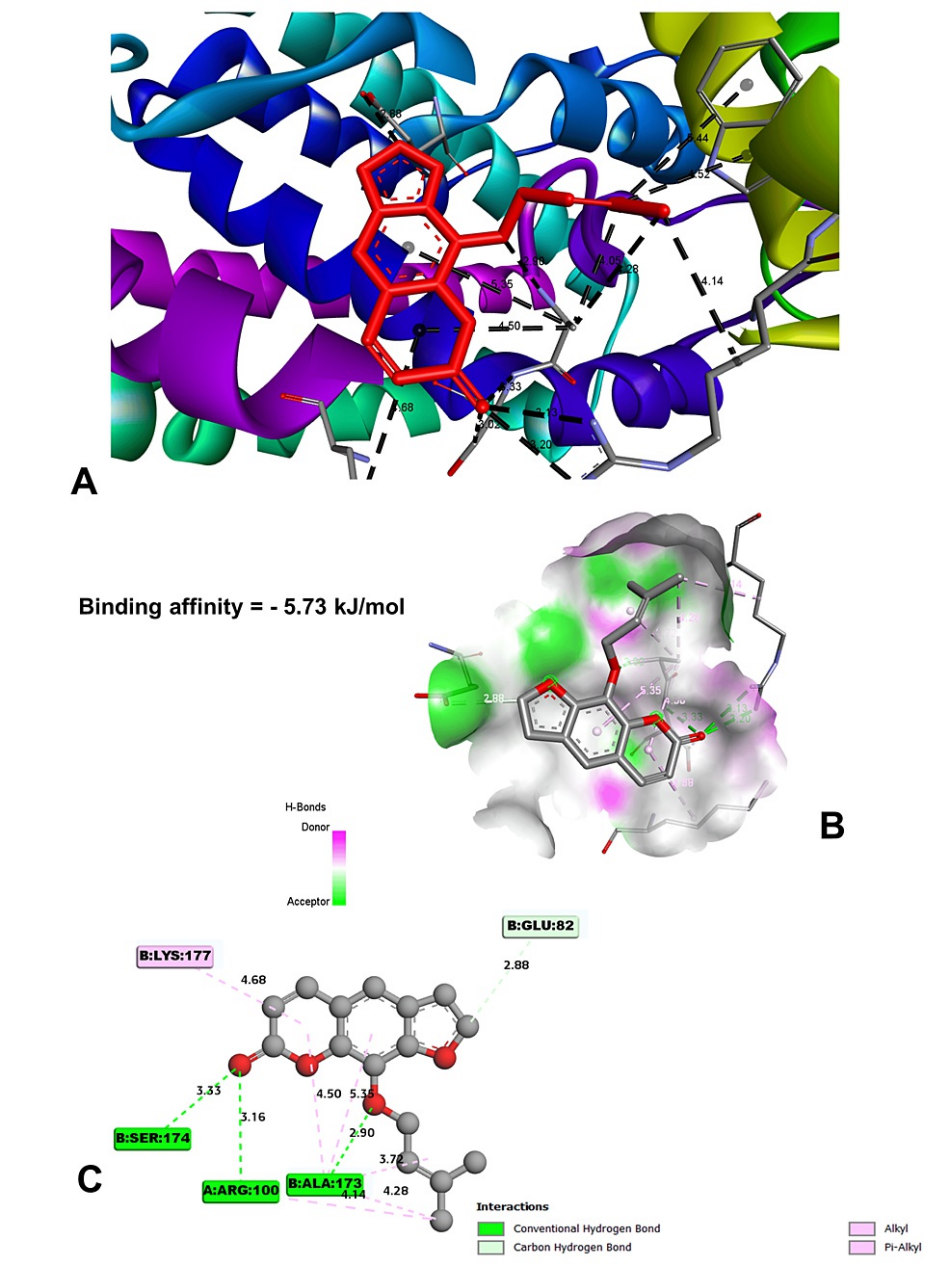
Molecular docking between imperatorin and HO-1 receptor (A) Binding orientation of heme oxygenase 1 (HO-1) and imperatorin. (B) and (C) Three-dimensional and two-dimensional representations of interactions between imperatorin and receptor molecule. (Image generated by BIOVIA Discovery Studio and Chimera software)

**Figure 6:**
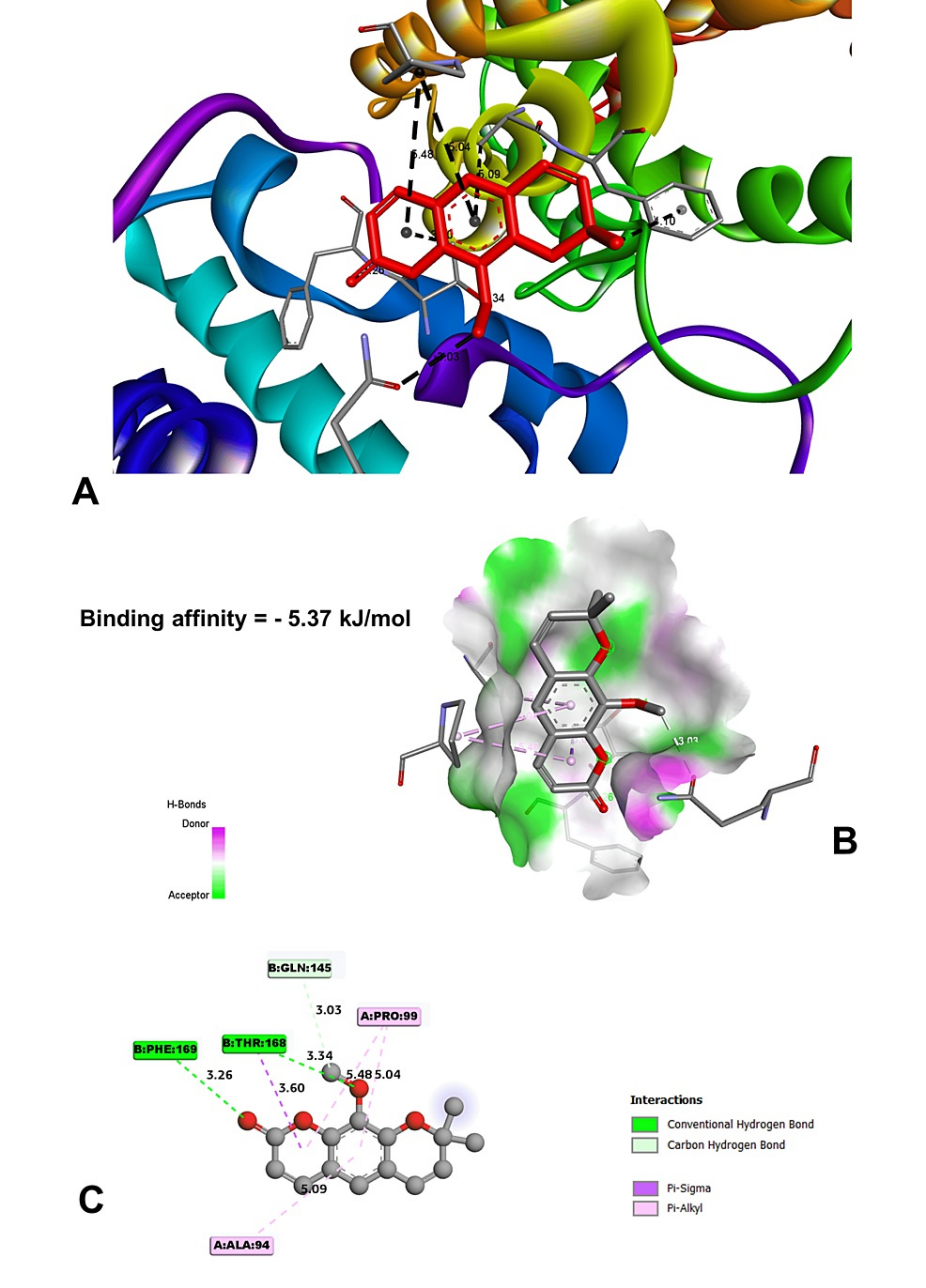
Binding posture of the luvangetin molecule in the HO-1 receptor (A) Target site of luvangetin in heme oxygenase 1 (HO-1). (B) Three-dimensional molecular interaction of luvangetin with the receptor. (C) Two-dimensional molecular interaction of luvangetin with the receptor. (Image generated by BIOVIA Discovery Studio and Chimera software)

**Figure 7:**
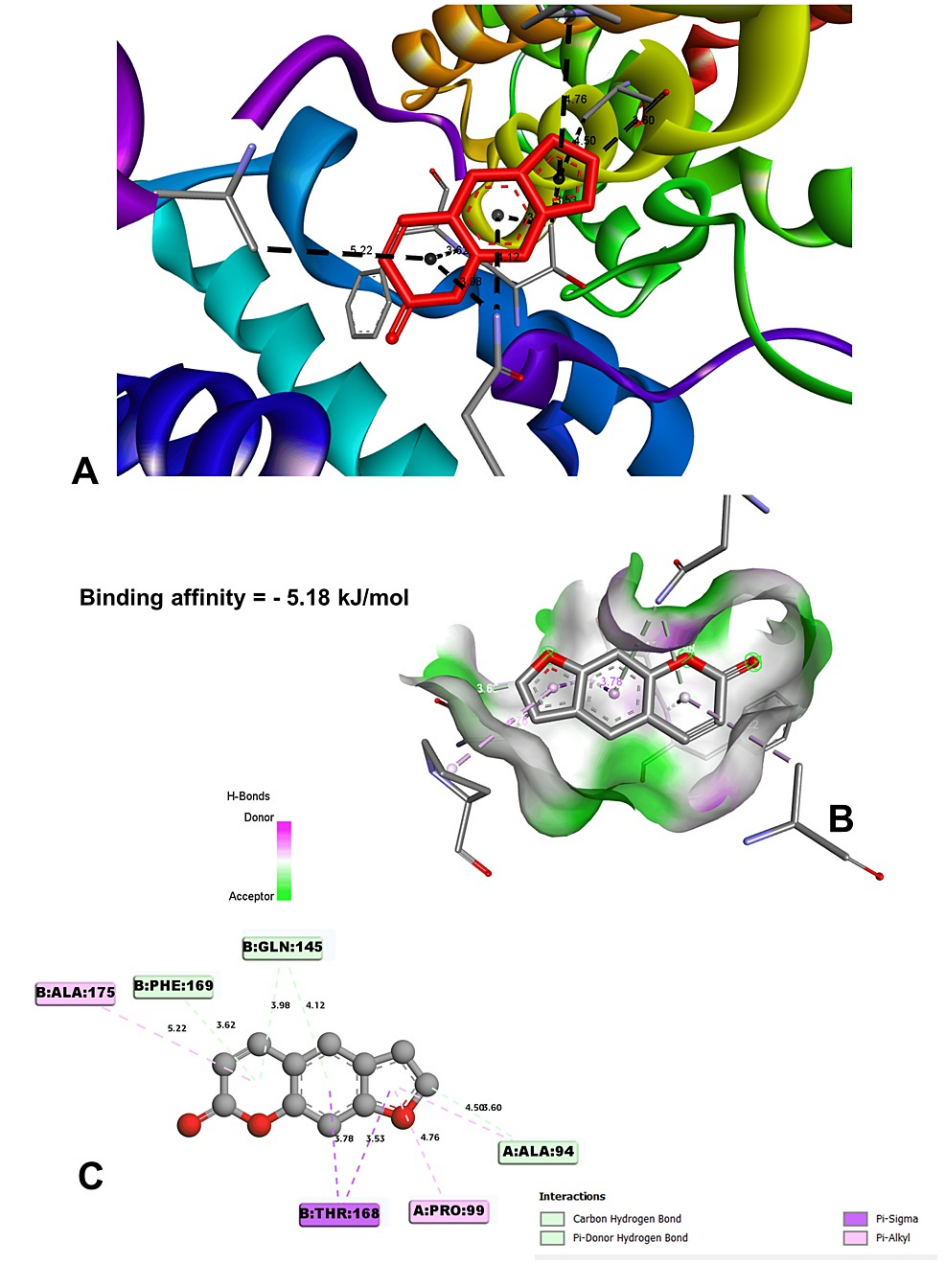
Molecular conformation study of psoralen and the HO-1 receptor (A) Psoralen binding site in heme oxygenase 1 (HO-1). (B) Three-dimensional interaction of psoralen with the receptor. (C) Two-dimensional interaction of psoralen with the receptor. (Image generated by BIOVIA Discovery Studio and Chimera software)

**Figure 8:**
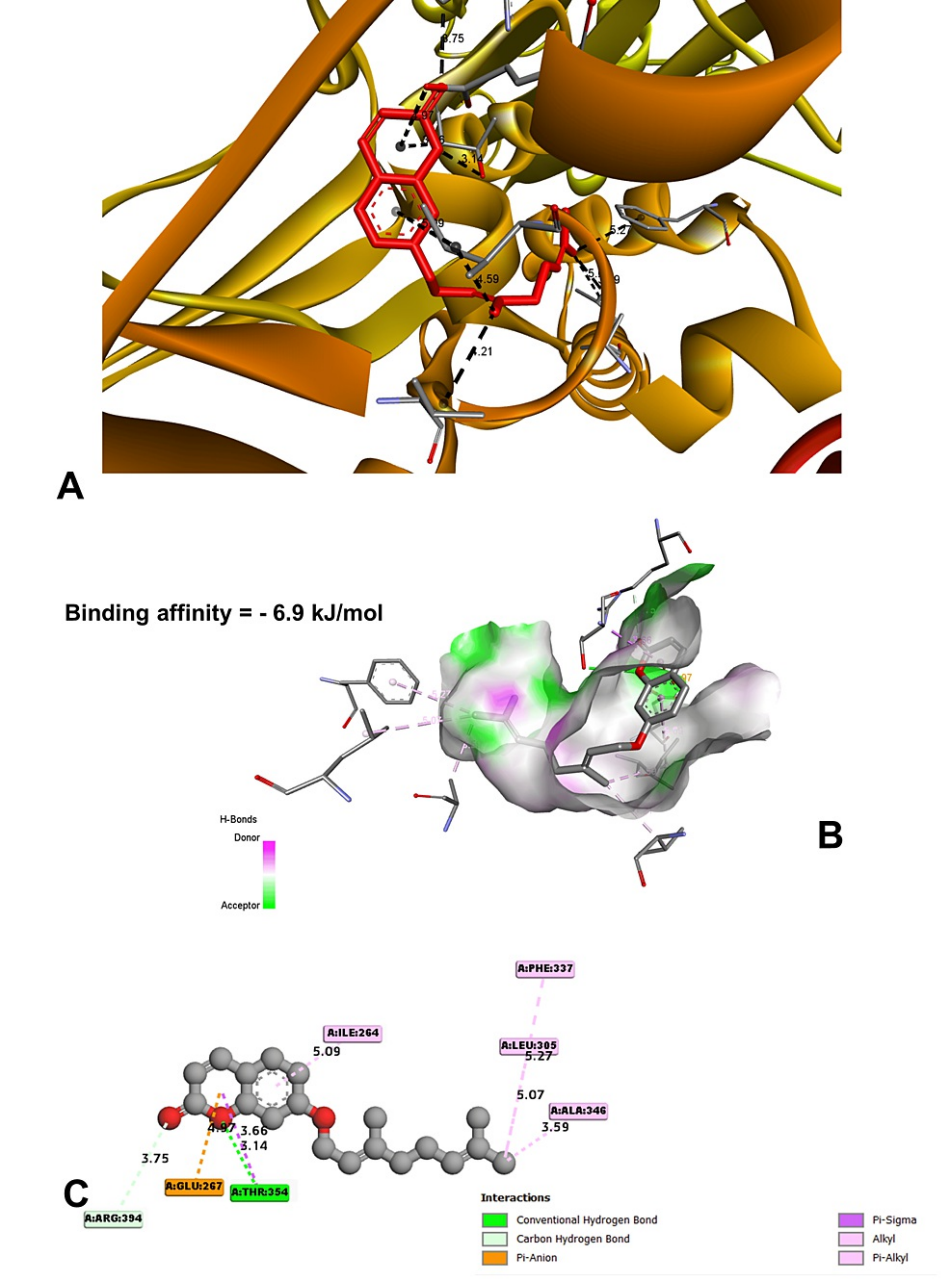
Characterizing the binding orientation of auraptene with xanthine oxidoreductase (A) Location of auraptene binding within xanthine oxidoreductase. (B) Three-dimensional interactions of auraptene and the receptor. (C) Two-dimensional interactions of auraptene and the receptor. (Image generated by BIOVIA Discovery Studio and Chimera software)

**Figure 9:**
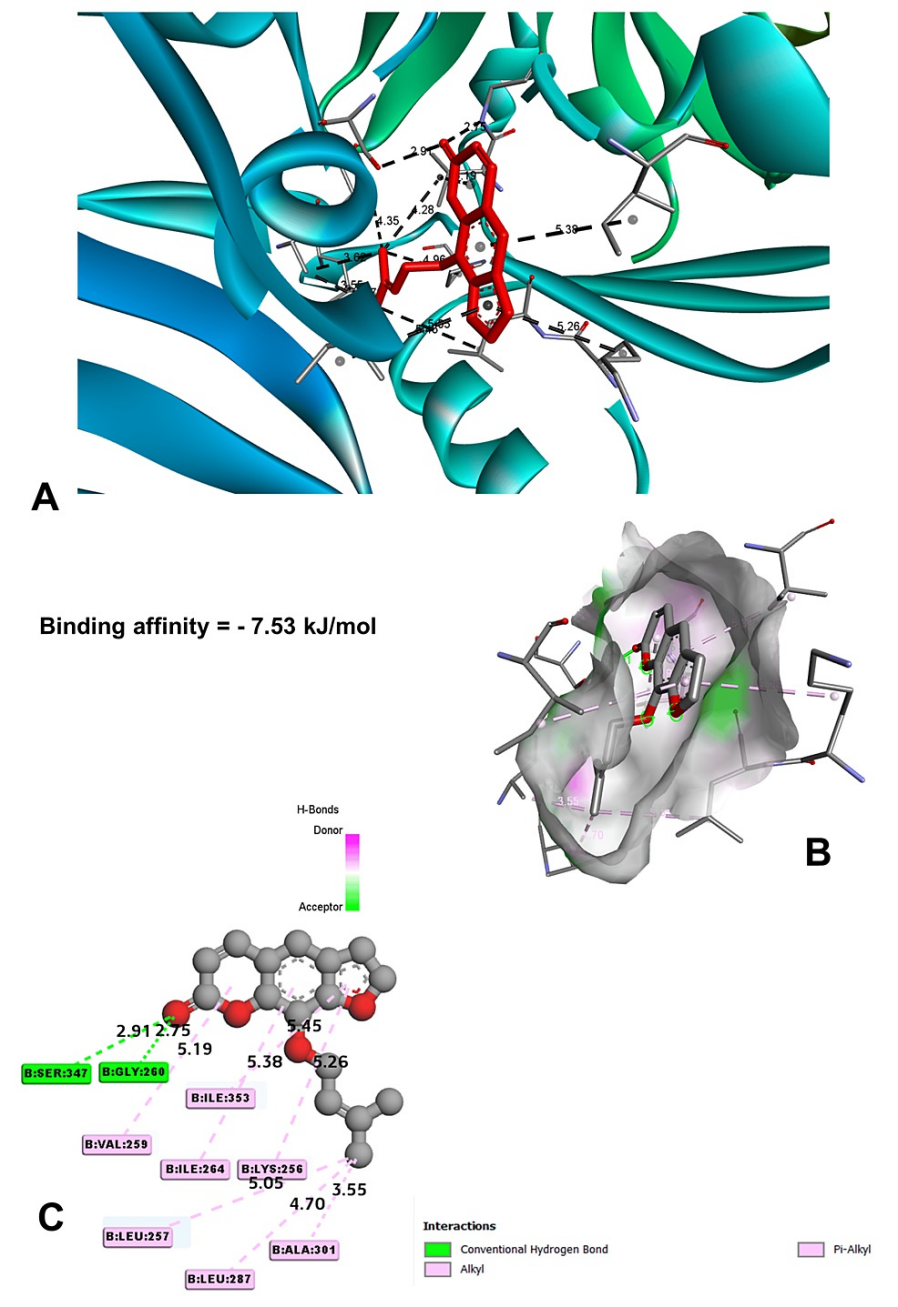
Binding conformation of imperatorin molecule with xanthine oxidoreductase (A) Location of imperatorin's binding site in xanthine oxidoreductase. (B) Three-dimensional interaction between imperatorin and the receptor. (C) Two-dimensional interaction of imperatorin with the receptor. (Image generated by BIOVIA Discovery Studio and Chimera software)

**Figure 10:**
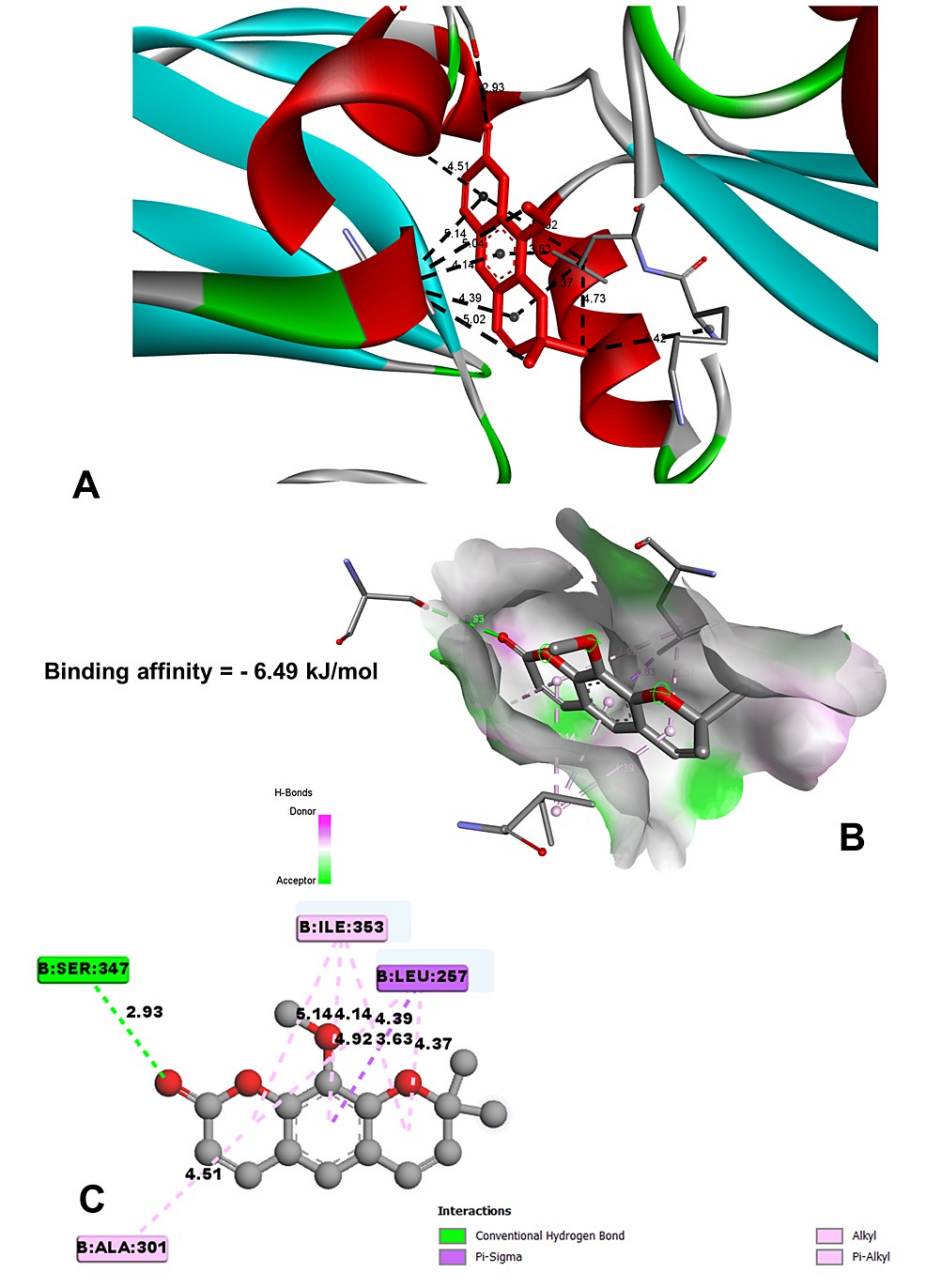
Conformational analysis of the luvangetin molecule (A) Luvangetin binding site in xanthine oxidoreductase. (B) Three-dimensional interaction of luvangetin with the receptor. (C) Two-dimensional interaction of luvangetin with the receptor. (Image generated by BIOVIA Discovery Studio and Chimera software)

**Figure 11:**
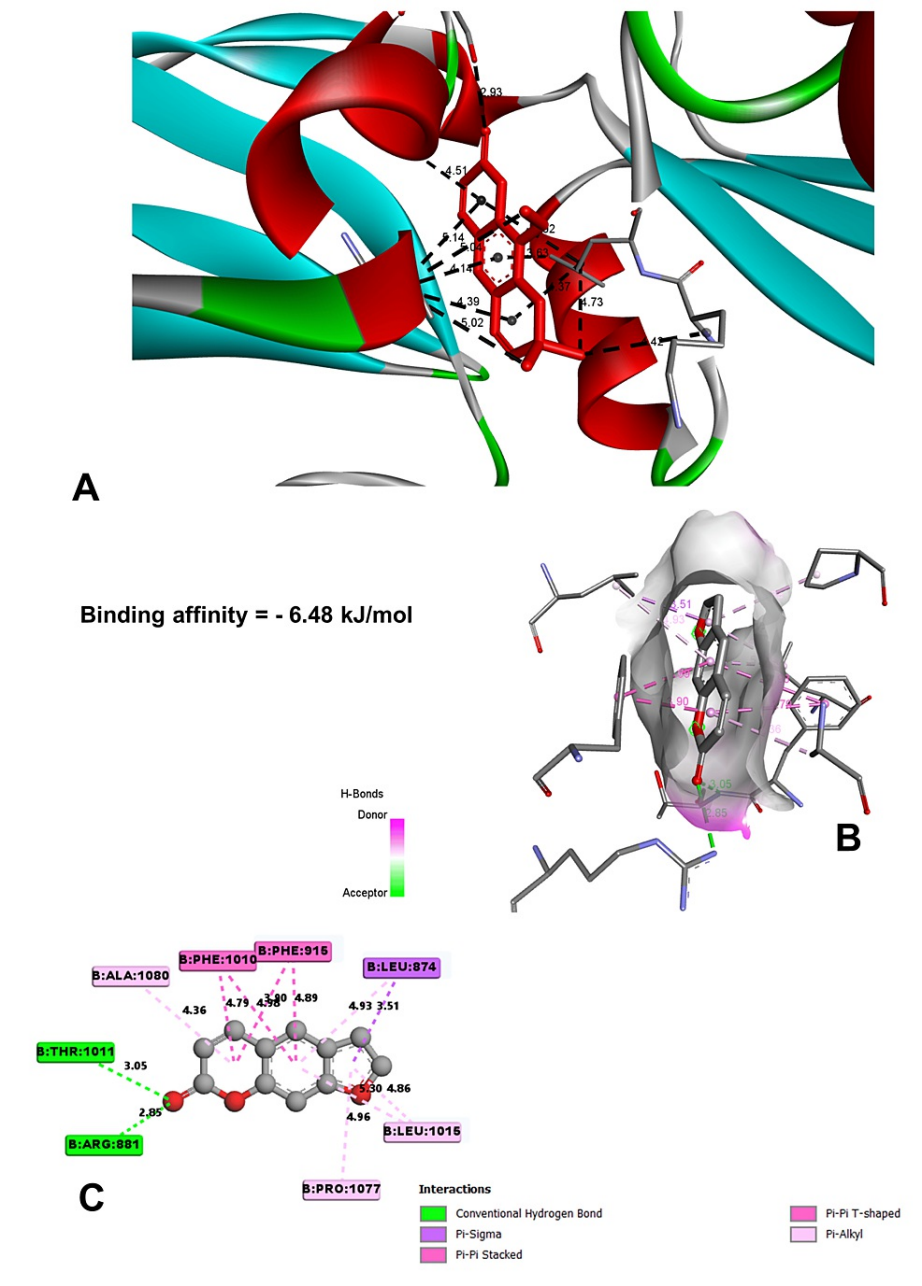
Binding conformation of the psoralen molecule. (A) Psoralen binding site in xanthine oxidoreductase. (B) Three-dimensional interaction of psoralen with the receptor. (C) Two-dimensional interaction of psoralen with the receptor. (Image generated by BIOVIA Discovery Studio and Chimera software)

In molecular docking analysis, two key enzymes, HO-1 and XO, and phytochemical components from *Aegle marmelos* were examined. Auraptene, for instance, exhibited a binding energy of -4.58 kJ/mol with HO-1, forming a robust hydrogen bond with GLY 143 at a distance of 3.5 Å (Figure 4). Imperatorin, displaying a compelling binding affinity with a binding energy of -5.73 kJ/mol, evinced notable hydrogen bonding engagements with ARG 100 (3.1 Å), ALA 173 (2.9 Å), and SER 174 (3.3 Å) (Figure 5). Luvangetin with a binding energy of -5.37 kJ/mol, forming hydrogen bonding interactions with THR 168 (3.3 Å) and PHE 169 (3.2 Å) (Figure 6). Psoralen, with a binding energy of -5.18 kJ/mol, demonstrated hydrogen bonds with GLN 145 (3.9 Å) and PHE 169 (4.1 Å) (Figure 7). These discernments underscore imperatorin's preeminence as the most potent binder to HO-1, followed by luvangetin and psoralen (Table 3). In XO, auraptene has a binding energy of -6.9 kJ/mol and forms hydrogen bonds with THR 354 (3.1 Å) and ARG 394 (3.7 Å) (Figure 8). Imperatorin demonstrated a remarkable affinity of -7.53 kJ/mol, establishing hydrogen bonds with GLY 260 (2.7 Å) and SER 347 (2.9 Å) (Figure 9). Luvangetin exhibited a binding energy of -6.49 kJ/mol, forming hydrogen bonds with SER 347 (2.9 Å) and LEU 257 (3.6 Å) in a complex manner (Figure 10). Psoralen had a binding energy of -6.48 kJ/mol, forming hydrogen bonds with ARG 881 (3.9 Å) and THR 1011 (4.1 Å) (Figure 11). Imperatorin was shown to be the most effective binder to XO, followed by auraptene, luvangetin, and psoralen (Table 4). The precise hydrogen bonding patterns revealed the intricate chemical mechanisms that govern their interactions with HO-1 and XO, highlighting their potential as therapeutic agents as anti-inflammatory and antioxidant compounds.

**Table 3:** Molecular docking results with interactive amino acids from human heme oxygenase 1 and phytochemicals from Aegle marmelos Distance: spatial separation between the ligand and receptor atoms

Compound	Binding energy (kJ/mol)	Amino acid	Distance (Å)	Interaction type	Interaction bond
Auraptene	-4.83	GLY 143	3.55557	Hydrogen bond	Carbon hydrogen bond
		ALA 28	3.48112	Hydrophobic	Alkyl
		ALA 28	3.6024	Hydrophobic	Alkyl
		HIS 25	4.28459	Hydrophobic	Pi-alkyl
		PHE 207	4.79336	Hydrophobic	Pi-alkyl
		PHE 214	4.91391	Hydrophobic	Pi-alkyl
Imperatorin	-5.73	ARG 100	3.13426	Hydrogen bond	Conventional hydrogen bond
		ARG 100	3.19518	Hydrogen bond	Conventional hydrogen bond
		ALA 173	2.9019	Hydrogen bond	Conventional hydrogen bond
		SER 174	3.33001	Hydrogen bond	Conventional hydrogen bond
		SER 174	3.02327	Hydrogen bond	Carbon hydrogen bond
		ALA173	4.28117	Hydrophobic	Alkyl
		TRP 101	4.52098	Hydrophobic	Pi-Alkyl
Luvangetin	-5.37	THR 168	3.34328	Hydrogen bond	Conventional hydrogen bond
		PHE 169	3.25598	Hydrogen bond	Conventional hydrogen bond
		THR 168	3.6003	Hydrophobic	Pi-sigma
		PHE 95	4.09696	Hydrophobic	Pi-alkyl
Psoralen	-5.18	GLN 145	3.97919	Hydrogen bond	Pi-donor hydrogen bond
		GLN 145	4.12297	Hydrogen bond	Pi-donor hydrogen bond
		PHE 169	3.61759	Hydrogen bond	Pi-donor hydrogen bond
		THR 168	3.53329	Hydrophobic	Pi-sigma
		THR 168	3.78276	Hydrophobic	Pi-sigma

**Table 4:** Molecular docking results with interactive amino acids from xanthine oxidase and phytochemicals from Aegle marmelos Distance: Spatial separation between the ligand and receptor atoms

Compound	Binding energy (kJ/mol)	Amino acid	Distance (Å)	Interaction type	Interaction bond
Auraptene	-6.9	THR 354	3.14022	Hydrogen bond	Conventional hydrogen bond
		ARG 394	3.75426	Hydrogen bond	Carbon hydrogen bond
		GLU 267	4.97405	Electrostatic	Pi-anion
		THR 35	3.66052	Hydrophobic	Pi-sigma
		ALA 346	3.58875	Hydrophobic	Alkyl
		PHE 337	5.26942	Hydrophobic	Pi-Alkyl
Imperatorin	-7.53	GLY 260	2.74767	Hydrogen bond	Conventional hydrogen bond
		SER 347	2.90852	Hydrogen bond	Conventional hydrogen bond
		ALA 301	3.54955	Hydrophobic	Alkyl
		ALA 301	3.6183	Hydrophobic	Alkyl
		ALA 302	4.35096	Hydrophobic	Alkyl
Luvangetin	-6.49	SER 347	2.92819	Hydrogen bond	Conventional hydrogen bond
		LEU 257	3.62725	Hydrophobic	Pi-sigma
		LEU 257	4.37441	Hydrophobic	Alkyl
		ILE 353	4.39393	Hydrophobic	Alkyl
Psoralen	-6.48	ARG 881	3.97919	2.85353	Hydrogen bond
		THR 1011	4.12297	3.04967	Hydrogen bond
		LEU 874	3.61759	3.51379	Hydrophobic
		PHE 915	3.53329	3.89591	Hydrophobic
		PHE 1010	4.7926	Hydrophobic	Pi-Pi T-shaped

## Discussion

Chronic inflammation and oxidative stress are key factors in many serious illnesses, creating significant health challenges worldwide. According to the World Health Organization (WHO), chronic diseases, many of which are associated with inflammation and oxidative stress, account for an estimated 60% of all deaths worldwide [17]. Furthermore, as populations age and lifestyle factors, such as poor diet and sedentary behavior, become more prevalent, the incidence of these conditions continues to rise. The increasing number of people affected by these conditions highlights the urgent need for new treatment approaches, especially those using natural products. With the therapeutic potential of natural products, researchers aim to develop safer, more efficacious treatments with fewer adverse effects compared to conventional pharmaceuticals. As these problems continue to affect people, finding effective new treatments becomes more and more important. This underscores the importance of research aimed at discovering beneficial treatments from natural sources.

The study aims to explore the antioxidant and anti-inflammatory properties of phytochemicals sourced from *Aegle marmelos*. In antioxidant assays, the methanolic tuber extracts of *Aegle marmelos* exhibited an exceptional absorption percentage of approximately 87.4%, outperforming the reference standard. In a previous study, in vitro assays confirmed its antioxidant and anti-inflammatory properties, supporting its traditional use by inhibiting protein denaturation, *Aegle marmelos* exhibited an IC50 value of 95.64 µg/mL, comparable to aspirin [18]. In another study, it was noted that the methanol extract of *Aegle marmelos* leaf exhibited 2,2-diphenyl-1-picrylhydrazyl (DPPH) scavenging activity compared to other extracts, with a percentage of 79.52 ± 0.35%. Similarly, the methanol extract showed greater inhibition of albumin denaturation (65.30 ± 1.07%) compared to alternative extracts. In comparison to previous studies, the current research holds an advantage in exploring the antioxidant and anti-inflammatory properties of phytochemicals sourced from *Aegle marmelos* [19]. The current study reveals that the tested sample demonstrated a notable absorption percentage of approximately 79%, highlighting its significant anti-inflammatory potential. This underscores the potent antioxidant activity of the extracts, indicative of their efficacy in scavenging free radicals and alleviating oxidative stress.

The antagonistic response of inhibitors toward enzymes or protein receptors does not indicate therapeutic efficacy [20]. Hence, ADME and drug-likeness studies play a vital role in drug development by assessing the feasibility of delivering inhibitors to biological systems. The majority of ineffective therapeutic studies typically utilize inhibitors that exhibit inadequate ADME properties, along with significant toxicity to the biological system [21]. In the current study physiochemical properties of key phytochemical constituents, including auraptene, imperatorin, luvangetin, and psoralen, were explored. These compounds displayed molecular weights ranging from 186.16 g/mol to 298.38 g/mol, indicative of their size and complexity. Imperatorin notably exhibited a higher number of aromatic heavy atoms (13) compared to other compounds, suggesting structural distinctions.

By predicting pharmacokinetic parameters, researchers can optimize drug formulations and delivery methods to enhance bioavailability and reduce variability in drug response among patients [22]. This optimization can lead to improved treatment outcomes and patient compliance. In this study, pharmacokinetic analyses revealed favorable properties for drug development, including high human intestinal absorption percentages ranging from 95.416% to 97.755% and moderate to high CaCo-2 permeability values ranging from 1.295 to 1.634. Favorable intestinal absorption ensures optimal drug delivery, improving bioavailability, therapeutic efficacy, and patient compliance while minimizing side effects and contributing to cost-effective healthcare. Although exhibiting low to moderate permeability across the BBB with logBB values ranging from 0.129 to 0.472 and poor to moderate permeability into the central nervous system with logPS values ranging from -2.556 to -1.714, these characteristics may mitigate potential central nervous system-related adverse effects.

Molecular docking studies are vital in drug discovery, aiding in rational drug design, target identification, lead optimization, virtual screening, elucidating drug-target interactions, and predicting drug resistance [23]. They expedite the development of safer and more effective therapeutics by simulating ligand-protein interactions and optimizing drug candidates. In this study, molecular docking simulations elucidated the interactions between the phytochemicals and two key enzymes, HO-1 and XO. Imperatorin emerged as the most potent binder to both enzymes, demonstrating binding energies of -5.73 kJ/mol with HO-1 and -7.53 kJ/mol with XO. These results underscore the therapeutic potential of *Aegle marmelos*-derived phytochemicals in combating inflammation and oxidative stress-related disorders, warranting further exploration in drug development endeavors.

Limitations and future studies

Despite the promising findings, a limitation of this study is the focus solely on in vitro assays and molecular docking simulations. While these methods provide valuable insights into the potential therapeutic effects of *Aegle marmelos*-derived phytochemicals, further in vivo studies are necessary to validate their efficacy and safety in living organisms. To address these limitations, future research should encompass comprehensive preclinical and clinical studies to evaluate the safety and efficacy of *Aegle marmelos*-derived phytochemicals in humans. Long-term randomized controlled trials are warranted to elucidate their therapeutic potential in managing inflammation and oxidative stress-related disorders.

## Conclusions

The study focusing on *Aegle marmelos* antioxidant and anti-inflammatory properties demonstrates its potential as a therapeutic agent. Notably, its exceptional antioxidant activity and significant anti-inflammatory potential underscore its efficacy in combating oxidative stress and inflammation. Understanding the pharmacokinetic properties of these compounds (auraptene, imperatorin, luvangetin, and psoralen) is crucial for optimizing drug formulations, enhancing bioavailability, and minimizing adverse effects. Molecular docking studies further elucidate their therapeutic potential by revealing their interactions with key enzymes implicated in inflammation and oxidative stress pathways. Imperatorin, in particular, emerges as a promising candidate, demonstrating potent binding to critical enzymes. These findings emphasize the importance of continued research into natural products like *Aegle marmelos*, offering insights into novel therapeutic avenues for inflammation and oxidative stress-related disorders.
